# Vaccination Perception and Attitude among Undergraduate Medical and Teacher Education Students at Charles University, Prague, Czech Republic

**DOI:** 10.3390/vaccines8010136

**Published:** 2020-03-19

**Authors:** Jiří Šálek, Alexander M. Čelko, Jana Dáňová

**Affiliations:** Department of Epidemiology and Biostatistics, Third Faculty of Medicine, Charles University, 110 00 Prague, Czech Republic; martin.celko@lf3.cuni.cz (A.M.Č.); jana.danova@lf3.cuni.cz (J.D.)

**Keywords:** vaccination, Czech Republic, vaccination perception, confidence, immunization, public health, education, undergraduate students, attitudes

## Abstract

This cross-sectional comparative study was designed to evaluate different opinions and their impact on vaccine confidence, as perceived by students of two different university programs (medicine and teacher education), as both of them play important roles in patient education, with the latter major shaping the skills of critical thinking. Multi-item, opinion-based, paper-and-pencil anonymous questionnaires were distributed among students of medicine and teacher education. Data were sorted and divided into two sets to be analyzed using logistic regression. Out of a total of 722 respondents, 386 were medical students and 336 were teacher education students. While most respondents said they were not in favor of alternative medicine, a significantly higher number of alternative medicine followers were teacher education students. The positive vaccination perception rate (PVPR) is not dependent on the behavioral factors of student respondents (irrespective of their major) but is largely affected by their attitude to alternative medicine. Fear of infection dramatically increased the PVPR (up to 6.7 times) in those who were versus were not afraid of getting infected or were not quite sure whether to fear it. Fear of side effects of vaccination clearly decreased the PVPR, by at least 84%.

## 1. Introduction

Vaccination is a method of active immunization playing a most important role in the prevention of infectious diseases; hence the decrease in morbidity and mortality since the 18th century.

At present, the Czech Republic is faced with a growing trend towards parental refusal of routine vaccination [1,2]. The year 2018 witnessed a major change in the state of vaccine confidence within Europe since 2015, as France, Greece, Italy and Slovenia became more confident in vaccine safety, while the Czech Republic, Finland, Poland and Sweden reported some disruption of confidence in this respect over the 3-year period. A recent study found a correlation between a general practitioner’s (GP) confidence and confidence among the general population [3].

Our cross-sectional comparative study was designed to evaluate the vaccination acceptance rate within two cohorts of students (majoring in medicine and teacher education), as both of them play important roles in patient education and, in the case of teacher education, in shaping of critical thinking, easier to develop using the explicit instruction approach than by imbedded instructional paradigms [4].

Previous studies have focused mainly on vaccine confidence and hesitancy in vaccination or even reluctance to vaccination in parents as well as physicians´ attitudes towards vaccination [5,6,7]. Only few studies have sought to map the attitudes of younger adults or undergraduate students to vaccination and vaccine-preventable diseases and were mainly focused on the topical issue of HPV (human papillomavirus) infections, as the vaccine against HPV is nowadays continuously under scrutiny [8]. Many studies have addressed the attitudes and knowledge of young people towards particular vaccines only. To date, minimal attempts have been made to map the general perception of vaccination in younger adults or undergraduate students [9].

Educational interventions are crucial to improving parents´ knowledge about vaccination, hence the surge in vaccination coverage of their children [10]. As healthcare providers play an important role in vaccination of the population, they need to be adequately prepared for this task during their undergraduate education. Practical training (clinical placements and other methods of hands-on training) has been significantly associated with the students´ better preparedness for clinical practice [11].

## 2. Materials and Methods

The present study was conducted in Prague, Czech Republic, as a multi-item, opinion-based, paper-and-pencil anonymous questionnaire survey. The questionnaires were distributed among medical students and those enrolled in teacher education programs during April 2019. The questionnaire was divided into several areas: personal data (year of birth, gender, field of study and study year), lifestyle and opinion-based questions related to vaccination. The study protocol was approved by the Ethics Committee of the Third Faculty of Medicine, Charles University in Prague. Questionnaires were addressed to medical and teacher education students of all years of study and distributed and collected personally by project team members. Collected data were computerized, sorted and analyzed by a biostatistician.

## 3. Results

### 3.1. Sample Size Determination

A total of 722 respondents (386 medical students and 336 teacher education students) were enrolled in the study. Although the number of medical students was slightly higher, the two groups could be considered approximately identical since the difference was not greater than 15%.

The sample size of 722 respondents was justified for logistic regression using 17 covariates [12], and, as the minimum required number was 425 respondents, the results of the secondary objectives can be regarded as generalizable.

### 3.2. Statistical and Analytical Methods

Factors were primarily evaluated as either ordinal or dichotomous variables, the former including year of study, nutrition, vaccination experience, fear of side effects or infectious diseases whereas gender, faculty (school), smoking and alternative medicine were dichotomous variables. The above factors were further divided into three categories: demographic (gender, faculty and year of study), behavioral (smoking, nutrition) and attitude (alternative medicine, vaccination experience, fear of side effects or infectious diseases).

Categorical quantities were evaluated by count and proportions including 95% CI. The survey reported only a single continuous variable (age) expressed as mean and median (including 95% CI and interquartile range). The quantity “age” was not further analyzed, as it corresponded to the year of study. The age difference between the two groups of students was evaluated using the parametric *t*-test.

Categorical quantities were compared using either Fisher’s exact test (for two variables) or the chi-square test (for more than two variables). Odds ratio was expressed as crude or adjusted using logistic regression. All tests were performed with a level of significance of α = 0.05 over a bilateral confidence interval.

Statistical analyzes were performed using Prism 8 biostatistical software (GraphPad Software, Inc., La Jolla, CA, USA) and STATA version 15.1 (StatCorp, Lakeway Drive, Texas, TX, USA).

### 3.3. Descriptive Analysis

No significant differences were found between the two groups of respondents (386 medical and 336 teacher education students). As only 59 (less than 9%) respondents failed to provide their year of birth, absence of data completeness can be considered marginal (<20%), with a minimal impact on potential bias.

Significantly more responses were provided by women than men in both groups. The distribution of respondents by the year of study was similar at both schools; most participants in the survey were first-year students (Table 1). There were comparable numbers of smokers in both groups. Survey participants, whether majoring in medicine or teacher education, answered most often that they consumed meals without any restrictions; other dietary options were rare. The distribution of responses by dietary habits was almost the same at both schools.

### 3.4. Analysis of Predictors and Study Objectives

As shown in Table 2, most respondents said they were not in favor of alternative medicine; however, a significantly higher number of alternative medicine followers were teacher education students.

While both groups of students perceived vaccination as an important tool of prevention, students planning a career in education were less likely to be sure (24%). An encouraging finding was that only 1.8% of all respondents found vaccination inappropriate; significantly more of them were teacher education students.

A negative experience with vaccination was reported more often by teacher education students.

Medical students were more often (40%) afraid of infectious diseases than their counterparts majoring in teacher education (24%); strangely enough, one in five would-be physicians and one in three would-be teachers claimed they did not worry about contracting an infection.

Most survey participants (significantly more medical students than teacher education students) were not afraid of any side effects after vaccination. The null hypothesis was rejected, confirming the alternative one under which students of teacher education had a lower vaccine acceptance rate (72%) than medical students (92%). This was demonstrated by a test strength >99.7% and a response error of less than 3.5%.

The positive vaccination perception rate (PVPR) was independent of behavioral factors of both groups of students. Fear of infections dramatically increased the chance of achieving the PVPR (up to 6.7 times) over those concerned about the possibility of contracting an infection or were not quite sure whether or not to fear it. Fear of post-vaccination side effects clearly reduced the chance of achieving the PVPR by at least 84%.

Two main predictors play a role in the PVPR in both groups of students—fear of infectious diseases and fear of adverse events. The other predictors were in agreement with the whole sample of respondents. Although there were more teacher education students who favored alternative medicine, surprisingly no significantly different PVPR was observed between the proponents of alternative and traditional medicine. It seems that positive vaccination perception rates among future teachers are generally lower than in future physicians.

## 4. Discussion

The survey attempted to estimate the differences in vaccine confidence, i.e., the positive vaccination perception rate (PVPR), between medical students and teacher education students. It has shown that confidence in vaccination is lower in the group of would-be teachers than among future physicians. A number of studies investigating vaccine confidence have been performed, focused mainly on adults and with parents as the main target group in particular. There is a multitude of factors playing a role in vaccine hesitancy in the general population including the level of education, socioeconomic status, mass media, different beliefs and attitudes based on cultural specifics [13]. Despite robust evidence supporting the benefits of vaccination as a preventive measure against infectious diseases, there is still a strong anti-vaccine movement making use of the internet and social media to shape public opinion in their favor [14].

Presentation of pro-vaccination information to adults only encourages their anti-vaccine sentiment [15,16]. Therefore, teaching children effectively could be the way to improve their health literacy and better understanding of infectious diseases and immunization. In our digital age, there are a host of options for delivering science-based information to children—comic books, videos, games, and so on [17].

Teachers should present balanced views and remain neutral while addressing controversial issues. However, as their presentation could still be biased, they should be discouraged from taking advantage of their position of authority to impose their subjective views on pupils and students alike [18].

A recent study suggested the best ways and approaches for the transmission of information to children to combat vaccine hesitancy in this “post-truth era” by proposing to involve education about the basics of immunization and critical thinking [19].

We can see a lower PVPR in the future teacher group, based on their subjective attitudes and, especially, greater fear of vaccination-related adverse events. While representative, our study has potential limitations to generalization of its results as it focused on two defined groups of students, thus vaccination perception and teaching about “controversial” vaccine-related topics remains a subject for future research, as does the comparison of these two particular cohorts of students with the general population.

## 5. Conclusions

This study strongly supports the assumption that vaccine confidence is lower among teacher education students. The fear of infections dramatically increased the chances of achieving positive vaccination perception in the medically educated group of students. It can be assumed that teachers of future generations will have a weaker attitude towards vaccination than future doctors, and this could negatively impact health literacy. Curriculum designers should consider integrating the importance of immunization in their education, presenting clear and evidence-based information.

## Figures and Tables

**Table 1:** Descriptive analysis of respondents.

Predictors			Number of Subjects						*p*-Value
			Total (*N* = 722)		Medicine (*N* = 386)		Education (*N* = 336)		
			*N*	Proportion (%)	*N*	Proportion (%)	*N*	Proportion (%)	
Demographic	Sex	Male	200	27.7 (24.5–31.1)	141	36.5 (31.7–41.6)	59	17.6 (13.6–22.1)	<0.0001
		Female	522	72.3 (68.9–75.5)	245	63.5 (58.4–68.3)	277	82.4 (77.9–86.4)	
		*p*-value		<0.0001		<0.0001		<0.0001	
	Faculty	Medicine	386	53.5 (49.7–57.1)					
		Education	336	46.5 (42.9–50.3)					
		*p*-value		0.0099					
	Year of Study	1	226	31.3 (27.9–34.8)	118	30.6 (26.0–35.4)	108	32.1 (27.2–37.4)	Not Applicable
		2	171	23.7 (20.6–27)	57	14.8 (11.4–18.7)	114	33.9 (28.9–39.3)	<0.0001
		3	146	20.2 (17.3–23.3)	60	15.5 (12.1–19.6)	86	25.6 (21.0–30.6)	0.0011
		4	47	6.5 (4.8–8.6)	34	8.8 (6.2–12.1)	13	3.9 (2.1–6.5)	0.0095
		5	132	18.3 (15.5–21.3)	117	30.3 (25.8–35.2)	15	4.5 (2.5–7.3)	<0.0001
		*p*-value		<0.0001		<0.0001		<0.0001	

**Table 2:** Positive vaccination perception rate (PVPR) and odds ratio (gross cOR and mutually adjusted aOR) for the whole sample of respondents.

Predictors		Number of Subjects			cOR	aOR	*p*-Value
		*N*	*n*	PVPR (%)			
Sex	Male	200	180	90 (85.0–93.8)	1	1	
	Female	522	417	79.9 (76.2–83.2)	0.44 (0.27–0.73)	0.84 (0.47–1.51)	0.559
Faculty	Medicine	386	354	91.7 (88.5–94.3)	1	1	
	Education	336	243	72.3 (67.2–77)	0.24 (0.15–0.36)	0.34 (0.20–0.58)	<0.0001
Smoker	Yes	73	62	84.9 (74.6–92.2)	1	1	
	No	649	535	82.4 (79.3–85.3)	0.83 (0.43–1.63)	0.64 (0.29–1.41)	0.269
Nutrition	No restriction	650	540	83.1 (80.0–85.9)	1	1	
	Patient diet	31	24	77.4 (58.9–90.4)	0.70 (0.29–1.66)	0.77 (0.27–2.16)	0.615
	Over-weight diet	25	21	84 (63.9–95.5)	1.07 (0.36–3.18)	1.65 (0.44–6.24)	0.457
	Alternative nutrition	16	12	75 (47.6–92.7)	0.61 (0.19–1.93)	0.64 (0.14–2.80)	0.550
Alternative medicine	Yes	122	77	63.1 (53.9–71.7)	1	1	
	No	600	520	86.7 (83.7–89.3)	3.80 (2.45–5.88)	1.85 (1.09–3.16)	0.023
Negative vaccination experience	No	673	571	84.8 (81.9–87.5)	1	1	
	Possible	27	17	63 (42.4–80.6)	0.30 (0.14–0.68)	0.82 (0.29–2.36)	0.716
	Yes	22	9	40.9 (20.7–63.6)	0.12 (0.05–0.30)	0.73 (0.21–2.54)	0.624
Fear of infectious diseases	No	189	139	73.5 (66.7–79.7)	1	1	
	Possible	299	238	79.6 (74.6–84)	1.40 (0.91–2.15)	1.30 (0.80–2.13)	0.290
	Yes	234	220	94 (90.2–96.7)	5.65 (3.01–10.61)	6.65 (3.12–14.18)	<0.0001
Fear of adverse events	No	616	543	88.1 (85.3–90.6)	1	1	
	Possible	73	41	56.2 (44.1–67.8)	0.17 (0.10–0.29)	0.16 (0.09–0.31)	<0.0001
	Yes	33	13	39.4 (22.9–57.9)	0.09 (0.04–0.18)	0.07 (0.02–0.21)	<0.0001
